# Lipome géant palpébral droit

**DOI:** 10.11604/pamj.2017.27.49.11137

**Published:** 2017-05-18

**Authors:** Abdoulaye Napo, Youssouf Fofana

**Affiliations:** 1 Institut d’Ophtalmologie Tropicale d’Afrique, Bamako, mali; 2 Service de Dermatologie, Centre National d’Appui à la Lutte contre la Maladie, Bamako, Mali

**Keywords:** Lipome géant, palpébral, œil

## Image en médecine

Les lipomes sont des tumeurs sous-cutanées uniques ou multiples, de consistance molle, compressibles, arrondies ou lobulées, mobiles sous la peau. Ils peuvent se localiser n'importe où, mais plus particulièrement sur le cou, le tronc et les membres. Il survient surtout à l'âge adulte, sans prédilection de race, ni de sexe, avec dans certaines études, une légère prédominance féminine. Le lipome est dit géant lorsque la pièce d'exérèse est supérieure à cinq centimètres. Nous rapportons le cas de Mr BK âgé de 35 ans qui a consulté pour une tuméfaction de la paupière supérieure droite évoluant depuis 2008. Depuis quelques temps le patient ressent une gène fonctionnelle de l'œil droit. A la palpation, c'est une masse indolore, mobile et mole et le reste de l'examen était normal. Nous avions évoqué trois hypothèses diagnostiques: un lipome le plus probable, un kyste et une tumeur royale. La tomodensitométrie et l'échographie mettaient en évidence des images de lipome. L'excision chirurgicale de la tumeur a permis d'extraire la graisse bien encapsulée et elle mesurait 5,5x4 cm. L'histologie de la pièce opératoire a confirmé la nature graisseuse de la tumeur. Les suites opératoires étaient simples avec correction des troubles visuels

**Figure 1:**
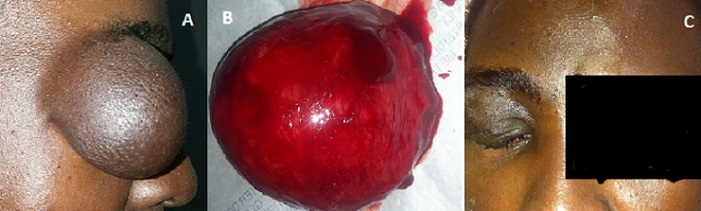
A) volumineuse masse palpébrale droite de consistance molle chez un patient de 35 ans faisant évoquer le diagnostic de lipome; B) masse graisseuse bien encapsulée après excision de la tumeur; C) suite post-opératoire

